# 

**Published:** 1844-08

**Authors:** 


### CONTENTS

OF NO. VIII.

### ORIGINAL COMMUNICATIONS.

### ESSAYS AND CASES.

ART. I.—On the Diagnosis of Phthisis Pulmonalis; being an Extract from a Lecture delivered at the General Dispensary, Louisville, Ky. By J. R. BUCK, M. D., Lecturer on Diseases of the Chest, in the Louisville Summer School of Medicine. - - - - 93

ART. II.—A Case of Abdomino-Intestinal Wound, successfully treated. By ANDREW R. KILPATRICK, M.D., of Woodville, Mississippi. - - - - - - - 100

ART. III.—A Case of Extensive Fracture and Depression of the Skull, in which the patient recovered without an operation. By D. C. SMYLEY, M.D., of Pleasant Hill, Alabama. - - - - - - - 104

ART. IV.—Foreign body in the Larynx—Laryngotomy. By C. J. THORNTON, M.D., of Henderson, Ky. - - 106

### REVIEWS.

ART. V.—A Practical Treatise on the Diseases of Children. By D. FRANCIS CONDIE, M.D., Fellow of the College of Physicians; Member of the American Philosophical Society; Honorary Member of the Philadelphia Medical Society, &c. - - - - - - - 108

ART. VI.—An Essay on the Blood in Disease. By G. ANDRAL, Professor of General Pathology and Therapeutics in the University of Paris. Translated from the

French by J. F. Meigs, M.D., and Alfred Stille, M.D. - - - - - - - - - - - - 123
Art. VII.—A Memoir of the Means of Modifying the forms of Living Bodies by Regimen. By M. Hippolyte Royer-Collard. Translated from the French of Dr. T. D. L. By John Barry, M. D., formerly Secretary of the Louisville Medical Society. - 141
Art. VIII.—The Cyclopædia of Practical Medicine. Edited by John Forbes, M.D., F.R.S., Alex Tweedie, M.D., F.R.S., and John Conolly, M.D. Revised, with additions, by Robley Dunglison, M.D. - 147

SELECTIONS FROM AMERICAN AND FOREIGN JOURNALS.

Brodie's Lecture on Diseases of the Tongue. - - - 148
Sulphate of Quinine in Yellow Fever. - - - - - 158
Dr. Drake's Letter. - - - - - - - - - 159
Quackery in England. - - - - - - - - - 162

ORIGINAL INTELLIGENCE.

Traveling Letters, No. II. - - - - - - - 163
Traveling Letters, No. III. - - - - - - - 176
Ipecacuan—Asthma. - - - - - - - - 179
Dr. Drake on the diseases of the South. - - - - - 181
Dr. Bell's Select Medical Library. - - - - - - 182
A Life of Accidents. - - - - - - - - 182
Medical Examiner and Western Medical Schools. - - 183
Louisville Summer School of Medicine. - - - - - 184
Medical Institute of Louisville. - - - - - - 184

